# A Values-Tailored Web-Based Intervention for New Mothers to Increase Infant Vaccine Uptake: Development and Qualitative Study

**DOI:** 10.2196/15800

**Published:** 2020-03-05

**Authors:** Amanda Dempsey, Bethany M Kwan, Nicole M Wagner, Jennifer Pyrzanowski, Sarah E Brewer, Carter Sevick, Komal Narwaney, Kenneth Resnicow, Jason Glanz

**Affiliations:** 1 University of Colorado, Denver Aurora, CO United States; 2 Kaiser Permanente Colorado Institute for Health Research Denver, CO United States; 3 University of Michigan, Department of Health Behavior & Health Education, School of Public Health Ann Arbor, MI United States

**Keywords:** immunization, parents

## Abstract

**Background:**

Vaccine hesitancy among parents leads to childhood undervaccination and outbreaks of vaccine-preventable disease. As the reasons for vaccine hesitancy are diverse, there is often not enough time during regular clinical visits for medical providers to adequately address all the concerns that parents have. Providing individually tailored vaccine information via the internet before a clinical visit may be a good mechanism for effectively allaying parents’ vaccination concerns while also being time efficient. Including tailoring based on values is a promising, but untested, approach to message creation.

**Objective:**

This study aimed to describe the process by which we developed a Web-based intervention that is being used in an ongoing randomized controlled trial aimed at improving the timeliness of infant vaccination by reducing parental vaccine hesitancy.

**Methods:**

Development of the intervention incorporated evidence-based health behavior theories. A series of interviews, surveys, and feedback sessions were used to iteratively develop the intervention in collaboration with vaccination experts and potential end users.

**Results:**

In all, 41 specific content areas were identified to be included in the intervention. User feedback elucidated preferences for specific design elements to be incorporated throughout the website. The tile-based architecture chosen for the website was perceived as easy to use. Creating messages that were two-sided was generally preferred over other message formats. Quantitative surveys identified associations between specific vaccine values and vaccination beliefs, suggesting that values tailoring should vary, depending on the specific belief being endorsed.

**Conclusions:**

Using health behavior theories, qualitative and quantitative data, and significant expert and end user input, we created a novel, Web-based intervention to improve infant vaccination timeliness. The intervention is based on tailoring messages according to each individual’s values and beliefs. This intervention is currently being tested in a controlled randomized clinical trial.

## Introduction

### Vaccine Hesitancy

Vaccination is widely recognized as one of the most effective public health interventions ever [1]. However, despite the well-established safety and effectiveness of vaccines, a growing number of parents are choosing to delay or forgo them for their children because of questions about vaccines’ necessity and safety (also sometimes because of firmly held religious or political beliefs). This phenomenon is referred to as *vaccine hesitancy* [2,3]. Vaccine hesitancy often results in undervaccination among children and has led to increasing numbers of vaccine-preventable disease outbreaks in the United States over the last two decades [4,5]. For example, measles, which was considered eradicated from the United States in 2000, caused infections among more than 2000 people between 2014 and 2018 alone, with the majority of cases occurring in individuals under- or unvaccinated against the virus [6]. Vaccine-preventable disease outbreaks are associated with significant cost and morbidity, and in some cases, even death [7]. Therefore, it is a public health priority to find interventions to mitigate this trend of parental vaccine hesitancy and childhood undervaccination [8]. This paper reports on the development of a novel, tailored, Web-based intervention to promote timely vaccination by addressing these issues. A more detailed description of the study design and protocol is available elsewhere [9].

### Study Rationale

Most interventions to increase vaccination developed thus far have focused primarily on correcting knowledge deficits, with the hypothesis that correcting these deficits will lead to improved attitudes and behaviors (ie, parents become less vaccine hesitant and thus are more likely to get their children vaccinated). Unfortunately, the majority of interventions based on this concept have not been effective at increasing vaccination rates [10,11]. This is because, as research has elucidated, parents’ vaccination decisions are multiply determined—based not *just* on their knowledge about the risk and severity of infectious diseases and the benefits and risks of vaccines but also on trust, emotion, values, past experience, access to health care, and peer influences [12,13].

Given the multiple determinants of vaccination decisions, novel intervention strategies that account for factors beyond knowledge deficits—such as personal values and emotions associated with parents’ individual concerns and barriers to vaccination—are needed [14]. One such approach to address these multiple issues is message tailoring. Message tailoring involves providing customized vaccine-promotion messages based on an individual’s unique beliefs, experiences, knowledge, and barriers to action [15]. Research on tailored messaging in multiple domains shows that by increasing the personal relevance of the information, people are more receptive to new information that may challenge their beliefs. Regardless of whether their *knowledge* of a given situation is altered by message tailoring, the technique is thought to work by lowering psychological resistance to information or suggested action that may counter what an individual initially thinks or believes [15]. Tailored messaging interventions have been shown to be effective for a number of health behaviors but have not been extensively tested for vaccine promotion [16,17].

In this paper, we describe the development of a Web-based, tailored messaging intervention used in a randomized controlled trial that is ongoing (ClinicalTrials.gov protocol number NCT02665013). This intervention, targeted to new and expectant mothers, was designed to promote vaccination by either reinforcing provaccination decisions among parents who are not hesitant to vaccinate or by decreasing vaccine hesitancy and thus increasing vaccination intention among parents who are vaccine hesitant. To do this, we developed our intervention based on evidence-based health behavior theories and included tailoring in both standard (ie, gender, primary vaccination beliefs, and concerns) and novel (personal values) domains. Although many additional factors could be novel targets for tailoring (trust, emotion, access to health care, etc), values were chosen as the potential new tailoring target for this intervention because, unlike beliefs, experiences, and barriers, which frequently change over time or by situation, values are believed to remain stable over the life course and across contexts [18-21]. Moreover, the behavior of vaccine hesitancy appears rooted in values as well as knowledge, skills, and self-efficacy [13,22-24]. Although there has been minimal research on how to use values to effectively promote vaccination, many researchers in the field recognize its potential importance [23,25-28]. Thus, a secondary goal in developing this values-tailored intervention was to begin to address the knowledge gap regarding the role values may play in creating messages effective for increasing maternal acceptance of vaccines. In addition, although fathers can be an important influence in childhood vaccination decisions, they were not included as the target for this intervention based on our previous research suggesting that mothers were the main vaccine decision makers in the study population of interest [29,30]. In this paper, we describe the overall intervention development process, with particular attention to how the novel values tailoring was developed.

## Methods

### Design Overview

The Web-based, tailored vaccine promotion messaging intervention was developed using a multiphase, iterative, user-centered design process. It was informed by behavioral theory, empirical data from maternal surveys and interviews, and expert and end-user input. When possible, end users’ opinions about intervention design were prioritized over those of the research team. For the intervention trial, we planned that mothers would be recruited to the website between the last trimester of their pregnancy and when their child was less than or equal to 2 months of age (the primary time for vaccination decisions to be solidified [30]). Mothers would then receive additional exposures to the website (with retailored information based on updated beliefs) at three additional time points between enrollment and infant age of 15 months. Vaccination timeliness would be assessed at age 200 days (ie, approximately 6 months) and 489 days (ie, approximately 15 months). Two versions of the website were developed for the randomized trial: a version with messages tailored to the individual participant based on personal characteristics (age, baby’s gender, and pregnancy status) and mothers’ vaccination beliefs and concerns, vaccination values, and intention to vaccinate (described in this paper), and an untailored version that was identical in appearance and content to the tailored version except for the tailored components (described elsewhere) [31].

**Figure 1:**
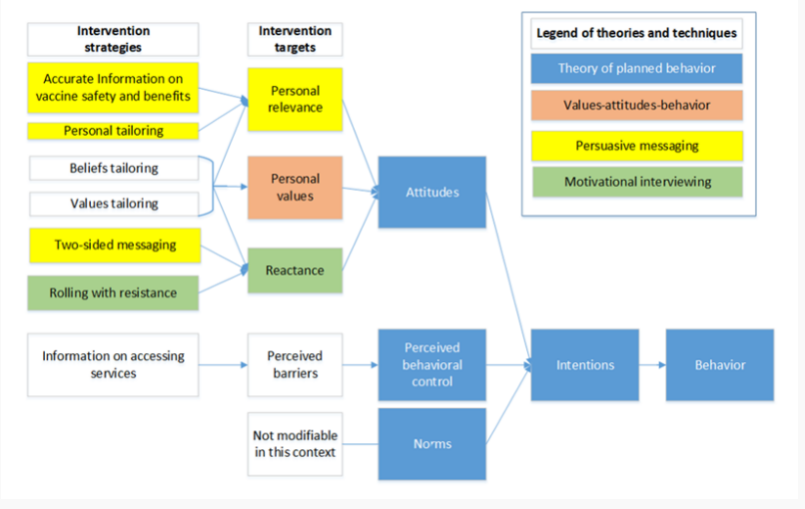
Conceptual model for a tailored messaging intervention to promote childhood vaccination.

Methods used to establish the tailored website’s architecture, content, and tailoring included the following:

1. A conceptual model reflecting empirically supported theories and intervention strategies for attitude and behavior change (Figure 1).
2. Developing informational content for the intervention using data from a previous intervention [32], the published literature, and end-user input.
3. Assessing the architecture of the intervention by evaluating an untailored prototype with usability testing and one-on-one interviews with potential end users of the website.
4. Iteratively developing and testing different message tailoring approaches using (a) survey data that assessed the relationships between maternal values and vaccination beliefs, (b) structured interviews with potential end users of the intervention on different types of message framing approaches in combination with tailored information, and (c) health communication expert and research team consensus.

### Conceptual Model

The theoretical framework for this intervention (Figure 1) was a hybrid of the Theory of Planned Behavior (TPB) [33] and the Values-Attitudes-Behavior (VAB) model [34]. According to the TPB, behavior (in this case, childhood vaccination) is directly influenced by intentions (intention to vaccinate), which are based on one’s attitudes, perceived norms, and perceived behavioral control. In the TPB, perceived behavioral control is a function of control beliefs (eg, beliefs that the decision to vaccinate is in the parents’ power to control) and attitudes are a function of behavioral beliefs (eg, beliefs that vaccinating one’s child will prevent infectious disease and will not harm the child). The VAB theory goes beyond the TPB to posit that personal values are factors influencing attitudes—this is the basis for our hypothesis that values-tailored messaging would be an effective behavior change target in the intervention. We also used select principles of motivational interviewing (MI) [35] and persuasive messaging [36,37] to inform intervention design, aiming to increase the personal relevance of the materials while minimizing reactance. Specifically for MI, the idea of *rolling with resistance* (not directly counteracting a person’s antivaccination attitudes for example) is believed to decrease a person’s reactance to receiving information that may be counter to their current beliefs [35,38]. Persuasive messaging in this context refers to making the information more personally relevant by tailoring the information to the person’s needs and more trustworthy by providing information that is perceived as accurate and balanced (two-sided messaging) with regard to potential *risks and harms* related to vaccination.

### Setting and Participants

**Figure 2:**
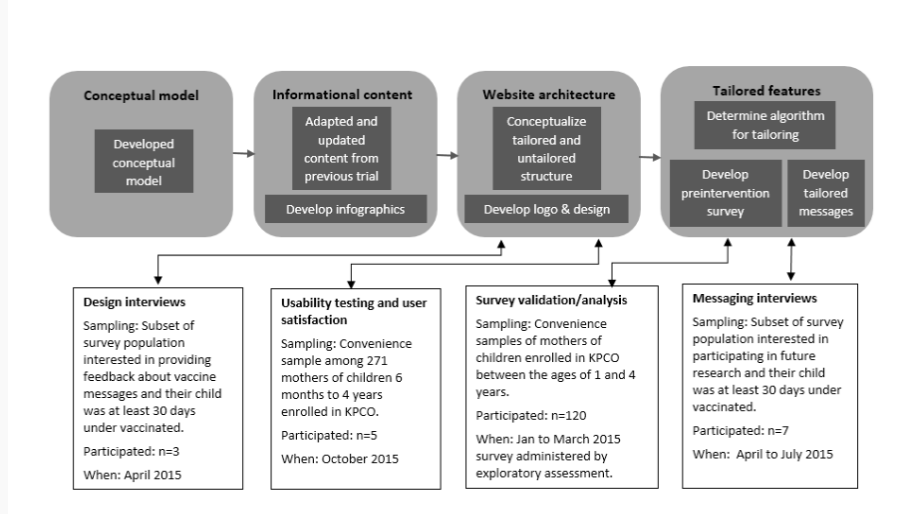
Study participant flow and activities.

The setting for this project was Kaiser Permanente Colorado (KPCO). KPCO is a managed care organization in the Denver metropolitan area that maintains an electronic health record with demographic, medical encounter, and vaccination data on all members. Between January 2015 and October 2015, various convenience samples (described below and in Figure 2) of mothers of young children currently enrolled at KPCO were recruited for the design and tailored message testing interviews, usability testing, and surveys.

### Developing Informational Content

Informational content for the website was developed by editing content adapted from an intervention used in a previous trial to reflect the most current vaccine information and recommendations [32], and developing new content based on emerging vaccination issues identified by vaccine experts and from vaccine questions received from parents in a previous trial [39]. When possible, content was contextualized to the local Colorado environment and health system (ie, reflected vaccines in the KPCO vaccine formulary) and was written at an eighth-grade reading level. All information presented was evidence based and included updated references from peer-reviewed journal articles and materials from the Centers for Disease Control and Prevention.

### Assessing and Finalizing the Architecture of the Intervention

#### Design Interviews, Usability Testing, and User Satisfaction

We solicited feedback from parents of children aged 1 to 2 years on preliminary color schemes, logos, and general architecture of the intervention using printed prototypes. These parents were recruited based on a prior indication of interest in participating in the intervention’s development when participating in the values survey described in the following sections.

On the basis of their feedback, we created an interactive, untailored, Web-based prototype to assess usability and user satisfaction. Using the electronic health record, we identified a random sample of 271 English-speaking mothers of children aged 6 months to 4 years currently enrolled in KPCO and not part of the previous study [32]. These individuals were recruited via email. Usability was assessed using the *think aloud* methodology, in which users provided verbal feedback as they completed specific tasks, such as logging on to the website and identifying specific types of information. User satisfaction was then assessed using the System Usability Scale (SUS) that includes 10 questions measured on a 5-point Likert scale [40]. SUS scores above 70 (total possible range 0-100) are considered passable. This feedback was incorporated to create the final version of the tailored (and untailored) websites.

### Iterative Development of Tailored Messages Incorporating Values and Framing

To create a personalized Web-based experience for each participant, the intervention was designed such that a preintervention survey would assess individuals’ values, beliefs, and vaccine hesitancy. The Web-based tailoring engine would then use this information to present corresponding tailored messages incorporated with evidence-based informational content about vaccination to each user. The process by which we developed and evaluated the values tailoring and message framing strategies is described in the following sections.

#### Values Survey

We first conducted a survey among KPCO mothers to identify which values were important for vaccination and whether values may have an impact beyond tailoring on beliefs. Described elsewhere [24], these data suggested that values had an influence on vaccination behavior that was separate but additive to the influence from beliefs and should therefore be included as a tailoring target. In this paper, we report our exploratory assessment of the associations between values and different beliefs and concerns as posited that some values would be better matched than others to some vaccination beliefs. Owing to time constraints of the study, we used email to recruit a convenience sample of mothers of children aged between 1 and 4 years who were currently enrolled at KPCO. Surveys were completed on the Web using REDCap (Research Electronic Data Capture, Nashville, TN, USA) and SurveyGizmo (SurveyGizmo, Boulder, CO, USA) survey systems. A US $30 incentive was provided. In addition to vaccination values, the survey also included measures of vaccination attitudes and beliefs and a global values measurement scale called the Schwartz Theory of Basic Human Values [21] to ascertain how values and attitudes relate to one another. Specific survey measures that were included are described in the following sections.

#### Vaccine Values

Parental values pertaining to childhood vaccination decisions (*vaccine values*) were measured using a scale the study team developed and assessed during this survey, called the Parental Vaccine Value Scale (PVVS). The PVVS is a 20-item scale that assesses 6 domains of values related to vaccination: security—disease prevention (valuing protecting one’s children from the harm of infectious disease; Cronbach alpha=.74), security—vaccine risk (valuing protecting one’s children from perceived harm of vaccines; α=.73), universalism (valuing protecting one’s community as a whole from the harm of infectious disease; α=.86), self-direction (valuing the process of gathering information to make an informed decision; α=.66), conformity (valuing the recommendations of experts and authority; α=.62), and tradition (valuing following the established norm in one’s religion or family; α=.79). These 6 domains were created to reflect a subset of global values in the Schwartz Theory of Basic Human Values, which was also examined during the survey [21,41,42]. The development and validation of the PVVS, including factor structure and alignment with the Schwartz global values, is described in a separate publication [24]. On the basis of the results of this analysis, the PVVS was chosen in favor of the Schwartz Theory of Basic Human Values scale as the tailoring target in the intervention. Values were assessed with a 4-point Likert scale that ranged from strongly agree to strongly disagree.

#### Vaccine Beliefs and Concerns

Beliefs and concerns about childhood vaccination were measured using a 10-item scale developed in a previous study [39], with three additional questions later added by the study team for the purposes of the project. A 5-point Likert scale (strongly agree to strongly disagree) was used to assess the responses.

#### Intention to Vaccinate

In all, 2 items that assessed mothers’ intention to vaccinate their newborn during the first year of life were used for message tailoring. These were based on performance of similar questions in a past study [39]. One item assessed how many of the 8 vaccines in the infant series a participant planned to have their infant receive with the following options: *none of the vaccines*, *some of the vaccines*, and *all of the vaccines*. The second item assessed when the mother intended to have their infant vaccinated with the following response options: *all on-time as recommended by my baby’s doctor* and *all or some later than my baby’s doctor recommends* (often referred to as a *delayed scheduled* or an *alternative schedule*). Combining these 2 items, mothers were categorized into 3 groups: refuses all vaccines, uses an alternative schedule (receives some or all vaccines but does not follow recommended timing), and full vaccine acceptor. The first 2 of these categories were grouped together to define *vaccine-hesitant* mothers when assessing vaccination outcomes at the end of the trial.

#### Analysis of Survey Data

From the survey data, we computed Spearman correlations between PVVS domain scores and the belief items. These results, combined with subsequent research team input, qualitative data, and expert opinion, were used to determine which values were associated with each specific belief or concern about vaccination and would therefore be included in the tailoring algorithm.

#### Interviews Exploring Message Framing and Values Incorporation

We considered several message framing options for the intervention messages. Using general information marketing approaches [43-45], we evaluated the potential usefulness of combining message framing with values framing approaches for the intervention messages. We considered the following approaches: 1) only values tailoring; (2) 1-sided messages, which present information about vaccines without acknowledgement of antivaccine arguments or negative aspects of vaccination [44]; (3) nonrefutational two-sided messages, which present both provaccination information and potential negatives (such as side effects) to the same extent (50% of information is pro and 50% is con) [44]; (4) *push* messages, which directly refute myths about vaccines and adopt a directive tone; and (5) *pull* messages, which provide information and invite the reader into a conversation or discussion about the topic while trying to encourage the desired action or behavior and encourage central processing or personal engagement in the content [37,43,45].

These various framing options were tested, along with the impact of different values and beliefs combinations, using structured interviews with 7 new mothers. The interviews took place between April and July 2015. Mothers were recruited from participants who took the survey described above and were eligible if they indicated in the survey an interest in providing feedback about vaccine messages, their child was at least 30 days undervaccinated as indicated in the medical record, and the mother had endorsed at least one of the six vaccine values domains in their survey (however, the messages tested were not necessarily matched to mothers’ most highly affirmed values, though this was attempted when possible). Potential participants were recruited using email and phone outreach. The interviews focused on assessing the participant’s acceptability of the messages and preferences between different formats. The interviews continued until saturation was reached. The participants received a US $50 gift card for their time.

**Figure 3:**
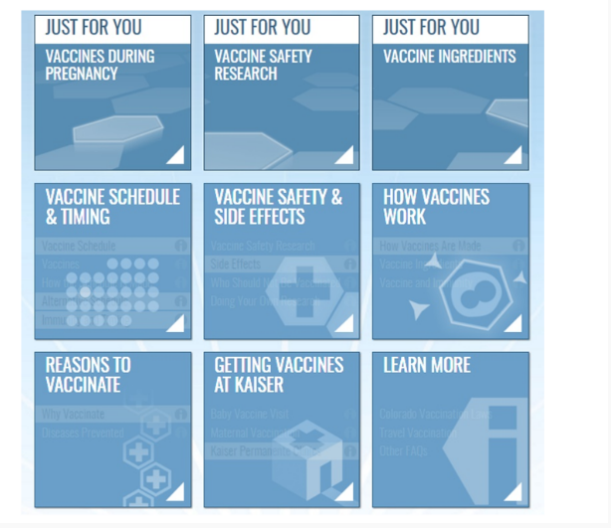
Architecture of the Final Tailored Intervention - Vaccines and Your Baby Home Page.

We designed the architecture of the intervention based on the concept of a *home page* consisting of different *tiles* representing different vaccination issues (Figure 3) as a central navigation point. We planned to have the most highly tailored information for the intervention presented in 3 *Just for You* tiles located prominently on the top of home page (Figure 3). These tiles represented the mothers’ three most pressing concerns or questions regarding vaccination. The intent of these tiles was to (1) facilitate the ease with which each parent could locate the content of highest interest, (2) increase mothers’ receptivity to information about their vaccine concern(s), (3) succinctly and credibly summarize key information about the concern, and (4) provide interested mothers with access to additional information about their concern. In the message framing interviews, 4 mothers were presented with up to 9 messages, which addressed as many as three featured concerns in the *Just for You* tiles. These messages included up to three messages tailored to value domains and up to three messages with different framing options (ie, a 3 × 3 factorial design; see Table 1 for examples of values tailoring messages). After viewing all the messages for an area of concern, mothers ranked the messages in order of their preferences and described their reasoning around their rankings. When a mother’s top choices aligned with their measured values, we considered the message a candidate for the final intervention. When mothers’ measured values and top ranked messages conflicted, we revised the messages to better align with the reasons that the mothers provided in the message framing interviews. Revised messages were further tested in subsequent interviews using the same interview format but with 3 different mothers.

**Table 1:** Examples of values-tailored messages. (Vaccines and Your Baby: Tailored Messages.)

Value	Topics	
	Alternative/delayed vaccine schedules message	Doing your own research on vaccines message
Security—disease prevention	Like many parents, your main goal is to keep your child healthy. The last thing you want is for your child to get an illness you could have prevented with a simple vaccine.	You’re the kind of person who will do everything she can to protect her baby from illnesses.
Self-direction	You’re not one to just do what other people tell you to do. You know your child better than anyone, and you have choices to make. You want to do your own research about vaccines. You don’t want him/her to get a disease. But you don’t want to put him/her at risk by getting vaccines.	You’re the kind of person who plays an active role in decisions about her baby’s health.
Security—vaccine risk	That’s a lot of needles (and a lot of tears)! You want to protect your child. But with so many vaccines at once, you’re concerned about exposing him/her to too many unnatural ingredients all at once.	You’re the kind of person who will do everything she can to protect her baby from pain or unnecessary medicines.

### Tailoring Components

#### Tailoring on Beliefs, Concerns, Hesitancy, and Demographics

Consistent with many previous tailored interventions and the TPB, we planned a priori to incorporate tailoring based on mothers’ beliefs and concerns, intention to vaccinate, and demographic characteristics, as described in the following sections.

#### Belief Tailoring

Of the 13 vaccination concerns assessed in the preintervention survey, those receiving an average score of 3 or less (some were composite measures, possible range of 1-5, with lower values corresponding to increasing antivaccine views) were considered a *qualifying concern* that could potentially be tailored on. This value was chosen as the cutoff for categorizing a concern as qualifying, given that it would capture participants with less-than-positive (ie, neutral or negative) beliefs about the topic. If more than three concerns met this threshold, mothers were asked to select their top three concerns that formed the basis of the *Just for You* tiles. If only three concerns were identified, they became the 3 *Just for You* tiles. Concerns beyond the top 3 selected by the mother were highlighted in the website’s base content (see Figure 3). If there were less than three concerns, up to 3 default *Just for You* tiles were featured in priority order: *The Vaccine Schedule*, *Baby Vaccine Visit*, and *Kaiser Permanente Clinics*. These topics were chosen as they were felt to have the least potential for raising new concerns among mothers without at least three concerns. For mothers with no vaccination concerns, *Just for You* content was tailored (first tile only) by providing positive reinforcement about their decision to vaccinate. The vaccine schedule tile was tailored based on the child’s age at the time the website was being viewed.

#### Intention Tailoring

We tailored content on vaccination intention based on responses to the 2 vaccine intention questions described earlier (how many of the 8 vaccines in the infant series a participant planned to have their infant receive and when the mother intended to have their infant vaccinated). These responses were combined, and mothers were categorized into 1 of 3 mutually exclusive groups for tailoring (as opposed to only 2 groups for the planned final vaccination analysis of the trial) that was incorporated into the content of the *Just for You* tiles: refuses all vaccines, uses an alternative schedule (receives some or all vaccines but does not follow recommended timing), and full vaccine acceptor.

#### Demographic Tailoring

Tailoring on personal characteristics, including the mother’s pregnancy status and child’s nickname, was incorporated throughout the website content.

## Results

### Developing Informational Content

In total, there were 17 general topic areas that the intervention addressed, divided into 41 specific content areas (Table 2). These 41 content areas were further grouped into 6 broad categories corresponding to the 6 *standard* tiles (ie, not the *Just for You* tiles) presented on the intervention’s home page (Figure 3). Furthermore, 4 of the 41 content areas were newly developed for the intervention. The remaining 37 were adapted from the previous intervention [39]. In addition, three newly developed, interactive infographics were also included on the following topics: herd immunity (included under the *Community Benefits* topic), antigen counts in the past and current vaccines (included under the *Vaccine Ingredient Types* topic), and disease rates before and after vaccines (included under the *Risk of Diseases* topic). Content areas included in the 6 home page tiles were identical in the tailored and untailored websites.

**Table 2:** Topic areas (general tops: n=17; specific topics: n=41) covered in the intervention and corresponding tile on the home page (n=6).

General topic area of interest and specific topics covered within each		Name of corresponding tile on home page
**Vaccine schedule**		
	Recommended vaccine schedule	Vaccine schedule and timing
	Vaccines	Vaccine schedule and timing
	Safety of the schedule	Vaccine schedule and timing
	How the schedule is made	Vaccine schedule and timing
	Importance of vaccine timing	Vaccine schedule and timing
**Alternative schedules**		
	Reasons why we cannot recommend an alternative schedule	Vaccine schedule and timing
**Immunity and timing**		
	Baby’s developing immune system	Vaccine schedule and timing
	Parents’ main concerns about baby vaccines	Vaccine schedule and timing
**Vaccine safety research**		
	How vaccine studies are done	Vaccine safety and side effects
	Vaccine Adverse Event Reporting System, Vaccine Safety Datalink, Vaccine Injury Compensation Program—How vaccine side effects are reported	Vaccine safety and side effects
	Doing your own research^a^	Vaccine safety and side effects
**Vaccine side effects**		
	Mild/common side effects	Vaccine safety and side effects
	Serious/rare side effects	Vaccine safety and side effects
	Illnesses/conditions not currently linked to vaccines	Vaccine safety and side effects
	Who should be vaccinated?	Vaccine safety and side effects
**How vaccines are made**		
	Vaccine production	How vaccines work
	How Pharma works	How vaccines work
	Kaiser and Pharma	How vaccines work
**Vaccine ingredients**		
	Why some ingredients are needed for vaccine production	How vaccines work
	Vaccine ingredient types	How vaccines work
	Individual ingredients	How vaccines work
**Vaccines and immunity**		
	Natural versus vaccine immunity	How vaccines work
**Why vaccinate**		
	Community benefits of vaccination	Reasons to vaccinate
	Risk of diseases^a^	Reasons to vaccinate
	Worldwide risk of diseases^a^	Reasons to vaccinate
	Current outbreaks (measles and pertussis)^a^	Reasons to vaccinate
**Diseases prevented**		
	Vaccine-preventable diseases and vaccines given at Kaiser Permanente Colorado	Reasons to vaccinate
**The baby vaccine visit**		
	Before the vaccine visit	Getting vaccines at Kaiser
	During the vaccine visit	Getting vaccines at Kaiser
	After the vaccine visit	Getting vaccines at Kaiser
**Maternal vaccination**		
	Vaccines in pregnancy	Getting vaccines at Kaiser
	Safety of vaccines in pregnancy	Getting vaccines at Kaiser
**Kaiser Permanente clinics**		
	Transportation to Kaiser clinics	Getting vaccines at Kaiser
	Bus routes	Getting vaccines at Kaiser
	Kaiser clinic hours	Getting vaccines at Kaiser
**Colorado vaccine laws**		
	Colorado vaccine laws	Learn more
	Colorado vaccine exemptions	Learn more
**Travel vaccination**		
	Kaiser travel clinic	Learn more
	Vaccines and traveling abroad	Learn more
**Adolescent vaccination**		
	Adolescent vaccination	Learn more
**Other FAQs^b^**		
	Other FAQs	Learn more

^a^Newly developed for the intervention.

^b^FAQ: frequently asked question.

### Assessing and Finalizing the Architecture of the Intervention

#### Design Interviews

Participants included 3, white, non-Hispanic mothers of children aged between 1 and 4 years. All the mothers preferred design features that appeared to be associated with the health system. This included color palettes with blue and green and logos with clean lines, similar to KPCO website pages. On the basis of the user feedback, we selected a tailored (and matching untailored) website design that used an interactive tile-based homepage (Figure 3), menu navigation in multiple locations, layering of information using accordion style grouping, and pop-up information in select locations.

#### Usability Testing and Satisfaction Survey Results

Of 271 mothers contacted for usability and satisfaction testing sessions, we scheduled a convenience sample of the first 6 respondents for usability testing interviews. Mothers who completed the interviews (n=5) were all female, white, non-Hispanic, with at least some college education, and had income ranging from US $50,000 to US $90,000 or more. On the basis of their input, changes to the prototype architecture included adding submenus to the main page, using words in place of images to assist in identifying the schedule toggle feature, adding new links to content pages, and automatically closing content accordions to assist in reading and navigating long content areas. All but 1 user had SUS scores above the passable score of 70 (range 65-97.5), suggesting that the usability of the site was acceptable. Owing to the similarity in responses among mothers, additional interviews beyond these 5 were not undertaken.

#### Description of the Architecture of the Final Tailored Intervention

In the final intervention, mothers are first directed to an onboarding page designed to engage the participant in the website so that they are inclined to continue to view the content. It includes a welcome message, references the participant’s intention to vaccinate, explains the intent of the website and that the information presented will be tailored based on their survey responses, and visually depicts where they can find the tailored content.

Following this, mothers are taken to the main home page of the intervention (Figure 3). On this page, the bulk of message tailoring is received via the 3 featured *Just for You* tiles. Any additional topics of concern beyond the top three concerns are highlighted within the 6 interactive tiles on this page. All the tiles lead to additional content that is tailored on the participant’s personal characteristics.

### Iterative Development of Tailored Messages Incorporating Values and Framing

#### Values Survey Results

Table 3 shows the results of correlations between belief items and values. These results informed team discussions about which values might be most appropriate for framing each concern topic. A combination of these data, research team consensus, and results from the parent interviews (described in the following sections) were used to determine which values were available as tailoring targets for each of the vaccine beliefs and concerns.

**Table 3:** Correlations between values and beliefs corresponding to website topic areas. Correlations (r) are Spearman correlation coefficients.

Beliefs^a^	Associations with provaccine beliefs			Associations with antivaccine beliefs		
	Universalism, r	Conformity, r	Security (disease prevention), r	Security (vaccine risk), r	Tradition, r	Self-direction, r
Enough research	−0.249^b^	−0.202^c^	−0.126	0.339 ^d^	0.247 ^b^	0.261 ^b^
Disease risk and benefit	−0.423^d^	−0.257^b^	−0.311^b^	0.202 ^c^	0.199 ^c^	0.080
Too many, too soon	−0.139	−0.364^d^	-0.199 ^c^	0.411 ^d^	0.257 ^b^	0.365 ^d^
Natural immunity	−0.315^b^	−0.226^c^	−0.204^c^	0.259 ^b^	0.324 ^d^	0.106
Vaccine safety	−0.123	−0.173	−0.078	0.352 ^d^	0.360 ^d^	0.180
Do own research	−0.173	−0.180^c^	−0.221^c^	0.249 ^b^	0.178	0.505 ^d^
Vaccine ingredients	−0.251^b^	−0.347^d^	−0.117	0.239 ^b^	0.240 ^b^	0.156
Autism	−0.205^c^	−0.231^c^	−0.092	0.338 ^d^	0.358 ^d^	0.164
Vaccine risk versus benefit	−0.186^c^	−0.115	−0.343^d^	0.474 ^d^	0.344 ^d^	0.333 ^d^
Combined risk/benefit	−0.319^d^	−0.176	−0.351^d^	0.412 ^d^	0.301 ^d^	0.257 ^d^

^a^Three additional vaccine topics were added based on expert feedback after this analysis was completed for a total of 13 belief topics.

^b^P<.01.

**^c^**P<.05.

**^d^**P<.001.

#### Interviews Exploring Message Framing and Values Incorporation

Of the 39 mothers contacted for interviews, 7 participated. All of them were mothers of children aged between 1 and 2 years.

Across topic areas, qualitative interviews assessing the general approach for framing messages revealed that two-sided messages were preferred by mothers compared with 1-sided, *push*, or *pull* messages. The two-sided message on side effects, presented as a table, was particularly well received. All mothers responded favorably to the intervention layout and reported that presenting both mild and rare side effects seemed *honest*. On the topic of alternative schedules (delaying or skipping certain vaccines), mothers generally (n=5) preferred the two-sided messages, largely because of the detail provided. The 2 mothers who followed an alternative schedule disliked the *black-and-white* messaging against this practice, believing this approach could sway mothers away from vaccinating at all. Finally, when 1- and two-sided messages were presented without values framing, mothers often (n=5) mentioned that values framing would improve the likeability of the message.

Among the values-framed messages, most (n=4) mothers preferred statements from *self-direction* values-tailored versions that acknowledged their *right to choose*. Mothers also responded favorably to the tailored versions of *security* that specifically addressed mothers’ motivation for keeping their child safe. Values-framed messages around conformity were the least preferred, with only 1 mother in the sample endorsing this value strongly. Values were not favored by any of the mothers for messages on the topic of side effects. These findings guided the team to adopt a two-sided messaging approach that incorporated values tailoring for topics of concern identified for mothers.

### Tailoring Components

#### Final Values Tailoring Algorithm

To build the final values tailoring algorithm, we used the results from the survey and interviews to identify the values or set of values that appeared impactful and relevant for each belief item assessed (Table 4). Any values exceeding the threshold of a 2.5 score (a score well above the *neutral* value of 2 on the 4-point scale used to assess values) that were also deemed impactful for that belief based on the survey and interview results were considered *relevant values*. We built an algorithm to randomly select one of these values and incorporate it into the corresponding *Just for You* tile. If the value has already been used in a previous topic area, another available value is selected at random from the available pool of values for that topic. If no additional values remain, all previously available values are made available for random selection. If the participant has no values that meet the threshold, a message without values framing for that vaccination concern is displayed. Values are generally incorporated into these tiles as *wrap-around* introductory sentences allowing for the core informational content on that belief to remain similar for each user. The same home page structure consisting of 9 tiles (Figure 2) was used for each retailoring.

**Table 4:** Values available for tailoring according to topic areas of the Just for You Tiles.

Title of the *Just for You* tile (content of corresponding general or specific topic areas potentially linked to tile)^a^	Applicable values					
	Security—disease risk	Security—vaccine risk	Self-direction	Conformity	Universalism	Tradition
Vaccine Safety Research (vaccine safety research)	x^b^	x	x	x	—^c^	—
Vaccine-Preventable Diseases (why vaccinate and diseases prevented)	x	x	x	x	x	—
Number and Timing of Vaccines (how the schedule is made, reasons why we cannot recommend an alternative schedule, immunity and timing, and vaccines and immunity)	x	x	x	x	—	—
Vaccine Ingredients (vaccine safety research and vaccine ingredients)	x	x	x	x	—	—
Vaccine Side Effects (vaccine safety research and vaccine side effects)	x	x	x	—	—	—
Doing Your Own Research on Vaccines (vaccine safety research and doing your own research)	x	x	x	x	x	—
The Immune System and Vaccines (immunity and timing, vaccines and immunity, and why vaccinate)	x	x	—	x	x	—
Vaccines and Autism (side effects and vaccine ingredients)	x	x	x	—	—	—
Vaccination Risks and Benefits (vaccine side effects, who should not be vaccinated, and why vaccinate)	x	x	x	—	x	—
Vaccines During Pregnancy (maternal vaccination)	x	x	x	x	—	—
The Role of Pharmaceutical Companies (how vaccines are made)	—	x	x	—	—	—
Alternative/Delayed Vaccine Schedules (reasons why we cannot recommend an alternative schedule, immunity, and timing)	x	x	x			—
Tips for Vaccinating at Kaiser (recommended vaccine schedule, baby vaccine Visit, and Kaiser Permanente clinics)	No values tailoring	No values tailoring	No values tailoring	No values tailoring	No values tailoring	No values tailoring

^a^Multiple General or Specific Topics related to several of the Just for You tiles. These tiles could include information on 1 or more general or specific topics depending on the user’s input.

^b^An 'x' in the table denotes that a given value is available to incorporate into the *Just for You* tile content.

^c^A '—' in the table denotes that the given value is not available to incorporate into the *Just for You* tile content.

## Discussion

### Principal Findings

In this paper, we described in detail how we developed a Web-based, tailored messaging intervention to address maternal vaccine hesitancy using an iterative development process and a mixed method approach. This intervention, which was developed using validated health behavior theories, is expected to be effective, engaging, and easy to use based on end-user feedback and pilot testing. Ultimately, the intervention included common tailoring elements such as demographics and beliefs as well as a novel tailoring target, personal values. In addition to examining tailoring targets, we also used user feedback to assess the potential impact of different message framing strategies in combination with the tailored elements. This was necessary as there is ongoing debate about the optimal messages framing strategy with regard to vaccine hesitancy and immunization [46-51].

### Application of Theory

Of the 4 health behavior theories and techniques used to inform the development of the intervention, the TPB has the most evidence for its applicability to vaccine decision making. Owing to this, we planned a priori to incorporate elements from this theory into our intervention and therefore did not focus any of our data collection described in this paper on these elements. Specifically, the intervention was tailored based on TPB constructs of attitudes, and when possible, elements of social norms were incorporated into the messages. The other remaining theories and techniques—the VAB theory and elements of persuasive messaging and MI—did not have a strong evidence base with regard to vaccine decision making at the time the intervention was being developed. However, the data collected in this study suggest that each is relevant to the vaccination decision. Specifically, results from our quantitative survey of values and from the qualitative interviews on message framing and values tailoring both suggest that certain values are more important than others with regard to vaccine decision making, and that some values are better incorporated with certain beliefs than others. A subsequent study done by our group examining the role that values play in mothers’ vaccine decision making further supports this notion [24]. Support for incorporating techniques from persuasive messaging into the intervention was also derived from the qualitative message framing and values tailoring interviews. The respondents clearly indicated that vaccine-hesitant parents strongly preferred messages that were perceived as *balanced*, where both positive and negative information (ie, two-sided messages) about vaccine safety or benefits was presented as these messages were deemed more trustworthy than messages that only conveyed one side of these issues. This point supports the concept in persuasive messaging that messages deemed as trustworthy are more likely to be reflected upon and more persuasive. Also supporting the importance of persuasive messaging was the finding from the message framing and values tailoring interviews that showed that messages tailored to the user were deemed more personally relevant. The design feedback interviews showing that mothers uniformly liked intervention architecture that clearly allowed a user to choose the specific information to view also support this concept. Evidence from our study for incorporating techniques from MI into the intervention is somewhat indirect in that mothers participating in the message framing and values interviews who were following an alternative vaccination schedule did not like *black-and-white* messages that argued against this practice. Such messages could be considered to counter the MI tenet of *rolling with resistance*. Another important MI tenet is that of using intrinsic motivation to effect behavior change. Evidence in support of this concept also comes from the message framing and values tailoring interviews showing that mothers generally preferred values-tailored messages to those not tailored to values. Further work by our group and others that occurred after our intervention was developed lends additional support to the important role that MI likely plays in motivating parents to vaccinate [52,53].

### Future Work

The next step in our study is to assess the efficacy of this intervention in a randomized controlled trial of KPCO expectant and new mothers. This trial (ClinicalTrials.gov protocol number NCT02665013) will examine the relative effect of the tailored versus untailored websites for their effect on timely infant vaccine utilization during the first 15 months of life. In addition, a number of secondary outcomes will also be assessed including whether the intervention modified maternal vaccination beliefs and concerns or vaccine hesitancy, and how any changes in these outcomes relate to vaccination values. This will allow for a more thorough investigation of the VAB model used as a basis for this study.

### Potential Importance of Values

Tailoring on values represents a novel, and potentially important, innovation incorporated into this intervention. A large body of literature in the social sciences provides compelling evidence that aligning educational messages with personal values can make information more salient and actionable [54-57]. The somewhat more limited number of studies examining values tailoring in the realm of health behavior change specifically further support this concept [58-61]. Recently, researchers interested in improving immunization delivery have begun to recognize the potential role of values tailoring in promoting vaccination [23,27,62]. Although there has been limited prior research examining the impact of values tailoring on vaccine acceptance, our own study [24] and that of others [63], which were completed after the study presented in this paper, have shown that differences in personal values are associated with variation in the acceptance of recommended vaccines. Taken together, values appear to be a potentially untapped tailoring resource that warrants further exploration. We believe results from the randomized controlled trial that will evaluate this intervention will add important and novel information to this growing body of research.

### Limitations

This paper should be considered in the context of some important limitations. First, when developing the various aspects of the intervention, we generally received input from only a small number of mothers, most of whom were white and non-Hispanic. Maternal input was not designed to be comprehensive. Rather, we opted to obtain in-depth information from a handful of mothers to provide more nuanced insight for optimizing the intervention and making it more relevant to potential end users, and we generally solicited mothers’ input until thematic saturation was reached (although we did not do a formal qualitative analysis). In addition, we focused much of the intervention development on the incorporation of values as a tailoring element, an approach to tailoring that is in need of further study. Moreover, although the study population providing input into the interventions’ design was diverse with regard to demographic characteristic such as race, ethnicity, education, and income, the population was relatively homogeneous with regard to insurance status (all had access to care) and primary language (we only gathered data from English-speaking mothers) and did not include fathers. As such, any impact of the intervention found in the upcoming clinical trial may not be generalizable to other populations that differ in these respects, and the intervention may need further refinement based on these characteristics. In addition, there are several items that previous research has shown as potentially important variables in the vaccination decision that could represent potential tailoring variables (education level, exposure to scientific controversy, degree of social networking, etc) [64,65] and were not included in our intervention. This was a purposeful decision to be able to isolate any potential impacts of values tailoring on vaccine uptake. However, these variables may need to be considered as tailoring targets in future iterations of the intervention. A notable strength of the project was the use of multiple methods to collect data (qualitative and quantitative), which may increase the validity of our findings.

### Conclusions

We used both qualitative and quantitative approaches and significant end-user input to develop a Web-based, theory-driven, tailored messaging intervention designed to address maternal vaccine hesitancy and subsequently improve uptake of infant vaccines. Our results suggest that elements from each of the 4 models and techniques incorporated into our conceptual model for the intervention were important. Specifically, results of qualitative user design and message framing interviews supported using several techniques from persuasive messaging and MI in the intervention. A quantitative survey on parents’ vaccination values, combined with qualitative data from the message framing interviews, supported the importance of the VAB model as a foundation of the intervention and supported the idea of using values as a novel tailoring variable. Elements from the TPB were not assessed directly in this study as there was already an evidence base supporting the importance of this theory in parents’ vaccine decision making. By incorporating elements from these four theories and techniques into the intervention, we believe it will be highly effective in changing mothers’ vaccination attitudes and behaviors. This hypothesis will be tested in a subsequent randomized controlled trial.

## Abbreviations

KPCO: Kaiser Permanente Colorado
MI: motivational interviewing
PVVS: Parental Vaccine Value Scale
SUS: System Usability Scale
TPB: Theory of Planned Behavior
VAB: Values-Attitudes-Behavior
